# The Effects of a Health Promotion Program Using Urban Forests and Nursing Student Mentors on the Perceived and Psychological Health of Elementary School Children in Vulnerable Populations

**DOI:** 10.3390/ijerph15091977

**Published:** 2018-09-11

**Authors:** Kyung-Sook Bang, Sungjae Kim, Min Kyung Song, Kyung Im Kang, Yeaseul Jeong

**Affiliations:** 1 Faculty of College of Nursing, The Research Institute of Nursing Science, Seoul National University, Seoul 03080, Korea; ksbang@snu.ac.kr (K.-S.B.); sungjae@snu.ac.kr (S.K.); 2 College of Nursing, Seoul National University, Seoul 03080, Korea; fattokki@snu.ac.kr (K.I.K.); Jeongfm1@snu.ac.kr (Y.J.)

**Keywords:** forests, mentors, health promotion, school-aged children

## Abstract

As problems relating to children’s health increase, forest therapy has been proposed as an alternative. This study examined the effects of a combined health promotion program, using urban forests and nursing student mentors, on the perceived and psychosocial health of upper-grade elementary students. The quasi-experimental study ran from June to August 2017, with 52 upper-grade elementary students from five community after-school centers. With a purposive sampling, they were assigned to either an experimental group (*n =* 24), who received a 10-session health promotion program, or to a control group (*n =* 28). Seven undergraduate nursing students participated as mentors. Running over 10 weeks, each weekly session consisted of 30 min of health education and 60 min of urban forest activities. Data were analyzed by independent *t*-test, Mann-Whitney U-test, paired *t*-test, or Wilcoxon signed rank test. General characteristics and outcome variables of both groups were homogeneous. The experimental group showed significant improvement in self-esteem (*p* = 0.030) and a significant decrease in depressive symptoms (*p =* 0.020) after the intervention, compared to the control group. These results suggest that forest healing programs may contribute to the spread of health promotion programs that make use of nature.

## 1. Introduction

In Korea, even school-aged children experience a high level of stress, anxiety, depression, and mental health problems [1]. In addition, the lack of physical activity due to sedentary lifestyles causes physical health problems such as an increase in obesity and decrease of physical strength [2]. Meanwhile, school violence has been increasing annually, and one possible cause of this problem is the lack of social interaction and emotional connection in modern society, caused by the increase in individualistic activities and computer use, and the decrease of contact and interaction with nature [3]. Kaplan and Kaplan [4] emphasized that the natural environment may be the best place to regain our attention and focus, and these benefits have also been shown to occur in children. Nature helps children to improve their psychological functioning [5], to cope with their problems, to think clearly, and to feel free and relaxed [6].

Recently, there has been considerable and increasing global attention towards using the forest environment as a place for recreation and health promotion. It has been noted that activities in the forest contribute to promoting physical and emotional well-being [7]. Forest healing refers to actions that enhance the health of the human body by utilizing various elements of the forest such as the landscape, phytoncide, water, wind, scent, and sound [8].

Countries such as the United Kingdom, Denmark, Sweden, and Finland are highly interested in situating children’s education in nature. Learning outside the classroom takes place in a variety of forms, including Forest School programs, outdoor sports, activities on school grounds, and visits to beaches and parks in the local community. The purpose and benefits of these experiences are also varied. Some focus on raising self-esteem and confidence; some on social skills, communication, and teamwork; and others on physical development and health [9,10]. Based on a systematic review, McCormick [11] reported that access to green space was associated with improved mental well-being, overall health, and cognitive development in children. Although some previous studies have reported positive effects of forest therapy on children’s mental health [12,13], nature-based intervention or education in Korea is still very limited.

In another aspect, a global trend shows differences in health status according to socioeconomic level, and various policies are being tried to reduce the gap. In Korea, one of the child welfare policies provides after-school care-giving at public “community centers for children” for low-income families with elementary- or middle-school students. However, the main services provided to children in these centers focus on educational or nutritional support, which does not provide integrated care in terms of the child’s physical, mental, and social aspects. In particular, there are no professional health-related services.

Therefore, in this study, we developed an integrated health promotion program including outdoor activities in a forest or urban park for the children attending community centers. The program was also expected to promote perceived health status and self-esteem, to reduce the symptoms of depression, to improve concentration through exposure to various resources of nature, and to enhance sociality through the use of group activities.

In addition, a special component of this study was that nursing students participated as mentors. When considering the developmental characteristics of school-aged children, it is possible to promote more active participation through forming relationships with college student mentors, who are more likely to encourage friendship and admiration. For the nursing students, participating in health promotion programs for children in vulnerable populations will be an opportunity to enhance their understanding of children’s developmental characteristics, and to form therapeutic relationships.

### Purpose of Study and Hypotheses

The purpose of this study was to develop a combined health promotion program using urban forests and nursing student mentors for vulnerable school-aged children and to evaluate the effects of this program on the perceived and psychological health of elementary school students in vulnerable populations. Specific hypotheses in this study were as follows:

1. The improvement in the perceived health status of children between pre- and post-program will be greater in the experimental group than in the control group.

2. Gains in self-esteem, peer relationships, and of sympathetic to parasympathetic autonomic balance and reductions in depression, attention deficit, and hyperactivity (ADHD) between pre- and post-program will be greater in the experimental group than in the control group.

## 2. Materials and Methods

### 2.1. Study Design and Participants

This quasi-experimental study, based on a control group pre-test–post-test design, was planned to develop a 10-session health promotion program using urban forests and nursing student mentors and to investigate its effects on elementary-school students in grades 4 to 6 at five community centers for children in Seoul. These community centers for children are public social welfare facilities that provide care services for vulnerable children. The children who use these centers are mostly from low-income families. Community child centers for children perform various important roles in community welfare, including offering protection and after-school education programs, providing healthy recreational and free-time programs, and linking children’s parents with the community [14].

**Figure 1:**
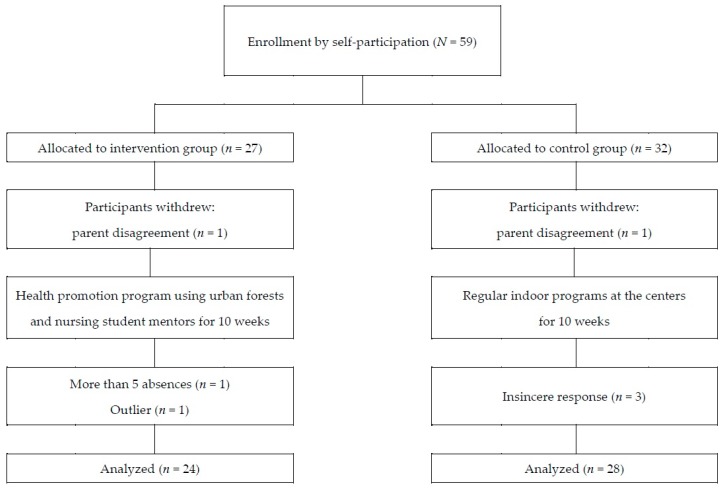
Recruitment of participants.

The study was approved by the Ethics Committee of the Institutional Review Board at Seoul National University (IRB No. 1707/003-001). Before beginning the study, participants and their parents were provided with a thorough description of the study’s objectives, experimental procedure, and measurement tools. Of the children and parents who decided to participate in this study, those who submitted their written consent were included in the study. Children who had medical contraindications to exercise by self-report (e.g., asthma, painful osteoarthritis) were excluded. A total of 59 students in grades 4 to 6 at five community centers participated in this study. With a purposive sampling, they were assigned to either an experimental group that was provided with a 10-session health promotion program with nursing student mentors (*n* = 27) or a control group (*n* = 32). Of the 59 children in grades 4 to 6 at the community centers for children, 57 agreed to participate in the study. The data of 52 children were finally analyzed, with the exception of insincere responses that were identified by being marked with the same numbers on all items of the questionnaire even though there were reverse questions (*n* = 3), participation in fewer than five sessions (*n* = 1), and those that were outliers and presented as an extreme value on the data distribution (*n* = 1; Figure 1).

### 2.2. Intervention

A combined health promotion program using urban forests and nursing student mentors based on a systematic review [15] and a needs assessment [16] was designed for children attending a community center. The 10-session health promotion program using urban forests consisted of lectures on physical and psychosocial health and forest experience activities. Each session allocated 30 min for the lecture and 60 min for the forest activities in the urban forests, which were 10 min away from each community center for children. The study developed a written program manual for the intervention providers to ensure the adherence to the intervention protocol and consistency throughout the program implementation. The program was provided once a week for 10 weeks, from June to August 2017. The control groups attended only the routine programs (e.g., supplementary learning such as math or English, reading, art) at their community center for children.

The program was conducted by the research team, all of whom were registered nurses whose majors were either child health or psychiatric nursing. One of them was a certified forest therapist, and one had completed the approved training course for forest therapy. During the sessions, two forest day camps were included; those were run by an external forest therapist. A total of seven nursing student mentors were involved in activities to facilitate the program and to promote the participation of children. The orientation was conducted before the start of the health promotion program to minimize any differences between the mentors. The major content of the orientation included an introduction of forest therapy, the specific contents and activities of the program, and the roles of mentors. Each mentor was responsible for three to four children, and two research team members supervised the mentors. In addition, the mentors shared significant matters that occurred during the program through the closed social network service (Kakao group talk), and their activities, feelings, and opinions were submitted anonymously through the Google questionnaire. The feedback from the forest therapist of the research team was provided and shared with all mentors. A social worker working at the community center for children accompanied the activities as an assistant for child safety. Table 1 shows the topics, contents, and time duration of each session.

### 2.3. Measurements

Participants’ height and weight were measured using an automatic stadiometer (BSM 370, Inbody Co., Ltd., Seoul, Korea). Heart rate variability (HRV) was also measured using a portable electrocardiograph (LXC3203, LAXTHA Inc., Daejoen, Korea). To minimize changes in autonomic nerves, HRV was performed after stabilization for at least 5 min, taking 5 min to complete the measurement. In this study, the HRV parameters were measured by a frequency domain analysis. The ratio of low frequency to high frequency (LF/HF ratio) in HRV was used to represent a measure of sympathetic to parasympathetic autonomic balance of the participants [17].

A pilot test using a self-report questionnaire was administered to elementary school students in grades 4–6 who did not participate in this research. As a result, it was confirmed that the students in upper grades did not have any difficulty in completing the questionnaire, which took about 20 min to complete at each time point. The specific instruments provided on the questionnaire were as follows.

Perceived health status. Perceived health status of participants was assessed by a single-sentence question: “How do you feel about your overall health condition?” It was rated as “very good (5 points),” “good (4 points),” “moderate (3 points),” “bad (2 points),” or “very bad (1 point).”

Self-esteem. Self-esteem was measured using the Rosenberg Self-Esteem Scale (RSE) [18] which includes 10 statements. Each statement is rated on a 4-point Likert scale with 4 = “strongly agree,” 3 = “agree,” 2 = “disagree,” and 1 = “strongly disagree.” The negative items were scored in reverse order. All items were added together with scores ranging between 10 and 40; a higher score means a higher level of self-esteem. At the time of tool development, the Cronbach’s alpha was reported as 0.88; the Cronbach’s alpha in this study was 0.85.

Depression. The Korean version of the Children’s Depression Inventory (CDI) [19] was selected for assessment of the participants’ depressive symptoms. There are 27 items quantifying depressive symptoms such as depressed mood, hedonic capacity, vegetative functions, self-evaluation, and interpersonal behaviors. Each item has a 3-point Likert scale as 0 = “absence of symptoms,” 1 = “mild or probable symptom,” and 2 = “definite symptom,” and a higher CDI score means a higher level of depressive symptoms. Han and Yoo [19] reported the Cronbach’s alpha as 0.81 for CDI in elementary and middle school students; the Cronbach’s alpha in the present study was 0.87.

Peer relationships. The peer relationship instrument [20] consisted of 20 questions for measuring the presence and reliability of friends, persistence of and adaptation to peer relationships, and communal life with friends. This instrument uses a 5-point Likert scale 1 = “not at all” to 5 = “very much,” and the higher the total score, the higher the level of the peer relationships. Kim [20] reported the Cronbach’s alpha as 0.94 for peer relationships for elementary school students, and the Cronbach’s alpha in the present study was 0.90.

Attention deficit and hyperactivity. The Korean version of Conners-Wells Adolescents Self-Report Scales (CASS-S, short form) [21] was used to assess the level of attention deficit and hyperactivity of the participants. This instrument includes 27 statements that evaluate behavior and cognitive problems, the level of hyperactivity, and ADHD indicators of respondents using a 4-point Likert scale from 1 = “not at all” to 4 = “it really is.” Higher scores indicate greater hyperactivity. As a result of the reliability test of CASS (S), the Cronbach’s alpha was 0.77; the Cronbach’s alpha in this study was 0.88.

### 2.4. Statistics

After normality tests using Kolmogorov-Smirnov test and Shapiro-Wilk test, an independent *t*-test or Mann-Whitney U-test was used to determine any differences in pre-test data between the experimental and control groups. Post-test data analyses were conducted using an independent *t*-test or Mann-Whitney U-test for comparison between groups, and a paired *t*-test or Wilcoxon signed rank test was employed for intragroup comparisons. The effect size was calculated using the effect size d for the parametric data and the effect size r for the non-parametric data [22]. Interpretations of d and r are as follows: d = 0.2 (small effect size), d = 0.5 (moderate effect size), d = 0.8 (large effect size), r = 0.1 (small effect size), r = 0.3 (moderate effect size), r = 0.5 (large effect size) [23,24]. SPSS for Windows Version 22.0 was used for all data analyses, and the statistical significance was set at *p* = 0.05.

## 3. Results

There was no significant difference between the experimental and control groups in the homogeneity test of general characteristics and outcome variables. The demographic characteristics and outcome variables of the groups are shown in Table 2.

Table 3 shows the comparison of pre- and post-test mean scores for the experimental and control groups. In the experimental group, self-esteem was significantly increased (*p* = 0.030, d = 0.47), and depression was significantly decreased (*p* = 0.020, r = −0.48) after the intervention. No statistically significant changes were found in the control group. This means that this program was partly effective in improving children’s psychological health.

## 4. Discussion

The aim of this study was to investigate the effects of a combined health promotion program using urban forests and nursing student mentors on the perceived and psychological health of elementary-school students. Previous studies have performed forest activities or green activities [25,26,27,28,29,30] or mentoring programs [31,32,33,34], but we expected positive reinforcement by not only using forest activities but also mentors in this study. As a result, self-esteem and depression were significantly improved in the experimental group, who participated in the health promotion program.

Previous forest activity studies conducted with children and adolescents reported that forest activities had positive effects on mental and physical health, including conditions such as ADHD, anxiety, depression, high blood pressure, and the lack of physical activity [11,35,36]. Also, a previous systematic review has reported that urban green space can increase physical activity levels, and access to urban green space also reduces stress and improves mood and the quality of life for people of all ages [37].

It has also been reported that green exercise has a positive effect on self-esteem and social interaction for adults [26]. Further studies that have examined the effectiveness of forest activity programs for children in community centers for children in Korea also reported improved children’s self-esteem [12,27]. In this study, after participating in the program, the self-esteem of the experimental group increased by 1.79 ± 3.79, whereas that of the control group decreased by 1.43 ± 4.73, indicating that the program improved self-esteem. However, some studies have reported that playing in nature has no significant effect on children’s self-esteem [25,30,38]. These researchers argue that children and adolescents may not receive the benefits of nature-friendly interventions because they do not experience the same level of connection with the natural environment as adults. Studies on the effects of forest camps and forest schools on improving psychological health outcomes, including self-esteem, have commented on the necessity of the direct use of the natural environment and suggested activities that use natural environments such as camping and horticulture [39]. In his book *Last Child in the Wood*, Louv [40], who had earlier attracted the attention of the world by emphasizing the need for contemporary children to be in contact with nature, has accumulated the positive effects of nature on children’s relaxation, self-worth, and self-esteem. He especially emphasized the importance of nature itself and did not focus on the structured program, but instead focused on giving them a chance to connect with and have free play in nature. Also, it was revealed that children who participated in camp demonstrated improved initiative and self-direction that transferred to their lives at home and in school. Therefore, children may need more time for direct interaction with or exploration in nature, as well as further structured interventions beyond simply playing in the forest, to have a more positive effect on self-esteem. In this study, the positive effect on self-esteem may be attributed to the direct interaction with nature activities and nursing student mentors in the intervention.

Also, by spending more time in and feeling the benefits of nature in a calm and natural environment, children’s nervous systems can relax from the resulting increase in parasympathetic nerve activation. While not found in this study, several previous studies with adults reported that forest activity leads to an activated parasympathetic nervous system, enhanced HF components of HRV, and lower LF/HF as a positive relaxation effect of forest therapy [41,42,43]. In previous studies that provided forest-based interventions, assessments of the effect were made immediately after the intervention. But in the present study, a post-test was performed one week after the last program was provided, which may have increased confounding factors. Therefore, further research is needed to consider the timing of measurement.

The differences in depressive symptoms within the group decreased in the experimental group (2.74 ± 5.55) and the control group (0.82 ± 4.44) in this study, but only that of the experimental group was statistically significant. In a systematic review of the effects of forest therapy on depression, forest therapy has been reported to be a new and effective intervention in reducing depression in elementary-school students as well as adults [15,44]. According to the meta-analysis, which evaluated the effectiveness of the forest-related programs, forest-related programs showed the greatest effect on depression (ES = 1.358) among psychological outcome variables, and self-esteem (ES = 1.269) and sociality (ES = 1.281) showed high effect sizes [45].

In this study, mentors seemed to function as an effective intervention tool, in addition to the forest activities. There was a positive effect of self-understanding, confidence, and achievement through the participants’ relationships with the mentors, which might reinforce the participants’ internal factor. This result shows a similarity to the results of previous studies that explored the effect of mentoring programs [31,34], in which the relationship between mentors positively affected the psychological health of vulnerable youths. According to the qualitative study of Akrimi, et al. [46], which explored the experience of school-aged children participating in a charity mentoring program with volunteer university-student mentors, the unconditional and supportive relationship between the children and mentors were commonly experienced. The developments of social-emotional, cognitive, and identity of the youth are accomplished based on their emotional connectedness with mentors and result in positive outcomes for the youth [33].

Previously, Kim [32] explored the competence of mentors using the Delphi analytic approach. Interpersonal skills, observation ability, and communication ability are necessary competencies for the mentors. With these skills, mentors can consider and respect themselves and others, sense mentees’ changes, and make accurate communication. These skills are emphasized in the field of nursing. For example, the Nursing and Midwifery Council [47] suggested communication and interpersonal skills as one of the standards for competence of registered nurses; therefore, educational nursing institutions should focus on educating their students to foster these abilities through the program. Thus, through the nursing students’ participation as mentors, they spontaneously formed supportive relationships with youth and educed children’s psychological and emotional development. An online survey and interviews were conducted for a qualitative study about the experiences of seven nursing students participating in this health promotion program. The qualitative study of the nursing students was undertaken at the same time as the evaluation of the children, but that the results have been reported separately [48]. As a result of qualitative research, this program was helpful for nursing students, as it provided opportunities for them to interact with and understand children.

Meanwhile, the peer relationship questionnaire item, used as an indicator of sociality, includes questions about the presence of friends, reliability, and continuity of friendship; it showed no significant difference between the groups. The questions about peer relationships mainly focus on the relationships with school friends, and thus the improvement of relationships with other children at the community center may not be reflected.

This study has some limitations. First, it was not possible to confirm whether the effect of the intervention was due to the mentoring or the urban forest activities because the health promotion program in this study utilized both urban forest activities and mentoring. Therefore, future studies will be required using a design to identify the individual effects of the forest activities and mentoring, and advanced analyses, such as mediation analyses. Such analyses will provide a clearer understanding of each component on the intervention. Second, it was a quasi-experimental study in which randomization was not used in place of intervention or assignment of the study participants. Community centers for the children participating in the study were selected based on accessibility and preference, which could lead to an imbalance or bias between study conditions and may have influenced subsequent analyses. Third, though both groups did not significantly differ at the baseline (as a result of lack of power), there seems to be a substantial difference in gender distribution among intervention and control condition. Particularly since girls might be more “vulnerable” to psychosocial health outcomes than boys [49,50], the influence of gender on the effectiveness of intervention cannot be ruled out. Fourth, consideration should be given to more diverse outcome variables such as emotions, which have been the most studied responses to green exercise [51]. As the health promotion program consisted of 60 min of activity after 30 min of lecture, there was not enough time allocated to being active in the forest, except for the time to move to the urban forest. It is also possible that the participants felt less interested in the program because they felt that the indoor lectures formed part of the classroom. Future research with a structured outdoor activity-focused program is recommended, and consideration should be given to encouraging the interest of the research participants. Also, more appropriate outcome measures might be required to identify the benefits of the program across multiple outcomes.

Despite these limitations, this program has visible strengths incorporating the feasibility of forest therapy as a health promotion program, which makes use of readily accessible urban forest parks, and the participation of nursing students as mentors, which might strengthen the psychological support for the participating children.

## 5. Conclusions

Our study showed that a combined health promotion program using urban forests and nursing student mentors significantly improved self-esteem and depression of elementary-school students in community centers for children. It is important that the forest healing program, which is growing in worldwide interest, has been applied to Korean children and that the perceived and psychological health effects have been grasped, which will contribute to the spread of health promotion programs using nature in the future.

## Figures and Tables

**Table 1:** The topics of program sessions.

Session	Lecture	Forest Therapy	Program Duration (hours)
1	Program orientation Meeting with mentors Pre-test		2
2	Understanding the relationship between physical and psychosocial health	Making a nickname for natural objects that resemble me Five senses experience in urban forest	2
3	Personal hygiene and eating habits	Physical activities in forest (I) -Forest walking and exercise	2
4	Internet overdependence	Playing with natural materials (I) -Making a paper fan with dry flowers and leaves	2
5	Forest day camp (I)		4
6	Self-emotion awareness and expression	Playing with natural materials (II) -Rock-paper-scissors game with natural materials -Water carrying using leaves	2
7	Self-esteem	Self-expression activities with natural materials Making a bracelet of retinispora beads	2
8	Communication and peer relationships	Physical activities in forest (II) -Cloth volleyball -Traditional play	2
9	Forest day camp (II)		4
10	Completion ceremony Discussion with mentors Post-test		2

**Table 2:** Homogeneity test of participants’ demographic characteristics and outcome variables during the pre-test (*N* = 52).

Characteristics/Variables	Categories	Exp. (*n* = 24) *n* (%) *M* ± *SD*	Cont. (*n* = 28) *n* (%) *M* ± *SD*	χ*^2^*/*t*/*Z*	*p*
Age (years)		11.83 ± 0.82	11.75 ± 0.97	0.33	0.741 ^†^
Gender	Male	8 (33.3)	14 (50.0)	1.47	0.225 ^‡^
	Female	16 (66.7)	14 (50.0)		
Grade	4th	10 (41.7)	11 (39.3)	0.09	0.958 ^‡^
	5th	8 (33.3)	9 (32.1)		
	6th	6 (25.0)	8 (28.6)		
Perceived health status		4.04 ± 0.95	3.68 ± 1.12	−1.31	0.191 ^§^
Self-esteem		31.42 ± 5.48	31.86 ± 5.06	−0.04	0.971 ^§^
Depression		12.26 ± 7.99	9.39 ± 7.27	−1.33	0.184 ^§^
Peer relationships		79.27 ± 12.34	78.54 ± 13.77	0.20	0.845 ^†^
Attention deficit and hyperactivity		15.09 ± 9.26	14.85 ± 11.57	−0.66	0.507 ^§^
LF/HF ratio		1.02 ± 0.58	1.67 ± 1.38	−1.83	0.068 ^§^

Exp.: experimental group; Cont.: control group; LF: low frequency; HF: high frequency; *M*: mean; *SD*: standard deviation; ^†^ *t*-test; ^‡^ χ^2^ test; ^§^ Mann-Whitney U-test.

**Table 3:** Comparisons of changes in outcome variables between the experimental and control groups (*N* = 52).

Variables	Group	Pre-Test	**Post-Test**	**Difference**	t or Z	*p*	ES d/r
		*M* ± *SD*					
Perceived health status	Exp.	4.04 ± 0.95	4.21 ± 0.83	0.17 ± 0.96	0.76	0.449 ^‡^	0.15
	Cont.	3.48 ± 1.12	3.86 ± 0.97	0.18 ± 1.22	0.62	0.538 ^‡^	0.12
Self-esteem	Exp.	31.42 ± 5.48	33.21 ± 4.35	1.79 ± 3.79	2.32	0.030 ^†^	0.47
	Cont.	31.86 ± 5.06	30.43 ± 5.03	−1.43 ± 4.73	−1.12	0.265 ^‡^	−0.21
Depression	Exp.	12.26 ± 7.99	9.67 ± 6.44	−2.74 ± 5.55	−2.32	0.020 ^‡^	−0.48
	Cont.	9.39 ± 7.27	8.57 ± 6.86	−0.82 ± 4.44	−0.63	0.531 ^‡^	−0.12
Peer relationships	Exp.	79.27 ± 12.34	78.96 ± 11.11	−0.43 ± 12.34	−0.16	0.875 ^†^	−0.03
	Cont.	78.54 ± 13.77	80.36 ± 13.40	1.82 ± 9.10	1.19	0.236 ^‡^	0.22
Attention deficit and hyperactivity	Exp.	15.09 ± 9.26	15.58 ± 11.99	0.22 ± 7.50	0.14	0.891 ^†^	0.03
	Cont.	14.85 ± 11.57	14.14 ± 9.77	−0.48 ± 9.85	−0.25	0.802 ^†^	−0.05
LF/HF ratio	Exp.	1.02 ± 0.58	1.05 ± 0.61	0.03 ± 0.71	0.19	0.849 ^†^	0.04
	Cont.	1.67 ± 1.38	1.45 ± 0.95	−0.22 ± 0.98	−1.20	0.241 ^†^	−0.23

Exp.: experimental group; Cont.: control group; LF: low frequency; HF: high frequency; *M*: mean; *SD*: standard deviation; ES: effect size; ^†^ paired *t*-test; ^‡^ Wilcoxon signed rank test.
